# Dynamic Similarity in Titanosaur Sauropods: Ichnological Evidence from the Fumanya Dinosaur Tracksite (Southern Pyrenees)

**DOI:** 10.1371/journal.pone.0057408

**Published:** 2013-02-25

**Authors:** Bernat Vila, Oriol Oms, Àngel Galobart, Karl T. Bates, Victoria M. Egerton, Phillip L. Manning

**Affiliations:** 1 Grupo Aragosaurus–IUCA, Paleontología, Facultad de Ciencias, Universidad de Zaragoza, Zaragoza, Spain; 2 Institut Català de Paleontologia Miquel Crusafont, Sabadell, Barcelona, Catalonia; 3 Departament de Geologia (Estratigrafia), Facultat de Ciències Universitat Autònoma de Barcelona, Cerdanyola del Vallès, Barcelona, Catalonia; 4 Department of Musculoskeletal Biology II, Institute of Aging and Chronic Disease, University of Liverpool, Liverpool, United Kingdom; 5 School of Earth, Atmospheric and Environmental Sciences, University of Manchester, Manchester, United Kingdom; 6 Department of Earth and Environmental Sciences, University of Pennsylvania, Philadelphia, Pennsylvania, United States of America; Ludwig-Maximilians-Universität München, Germany

## Abstract

The study of a small sauropod trackway from the Late Cretaceous Fumanya tracksite (southern Pyrenees, Catalonia) and further comparisons with larger trackways from the same locality suggest a causative relationship between gait, gauge, and body proportions of the respective titanosaur trackmakers. This analysis, conducted in the context of scaling predictions and using geometric similarity and dynamic similarity hypotheses, reveals similar Froude numbers and relative stride lengths for both small and large trackmakers from Fumanya. Evidence for geometric similarity in these trackways suggests that titanosaurs of different sizes moved in a dynamically similar way, probably using an amble gait. The wide gauge condition reported in trackways of small and large titanosaurs implies that they possessed similar body (trunk and limbs) proportions despite large differences in body size. These results strengthen the hypothesis that titanosaurs possessed a distinctive suite of anatomical characteristics that are well reflected in their tracks and trackways.

## Introduction

Sauropod dinosaurs represent one of the most diverse and evolutionary successful groups of terrestrial vertebrates that lived during the Mesozoic [1]. The clade comprises the largest vertebrates that ever walked on land, with adult individuals of some species estimated to have reached up to 30 metres in length [2], [3]. Sauropod locomotion has been a subject of intense research and in the last decades much insight has been gained from studies of ichnology and biomechanics [4]–[9] but very few studies have specifically examined the gaits of titanosaurs [10], [11]. This is surprising given the dominance of this group in Cretaceous faunas and their possession of a suite of anatomical features thought to underpin a shift in locomotor dynamics relative to more basal neosauropods [8], [12], [13]. Difficulty assessing titanosaur locomotion is largely attributable to their anatomy being poorly understood, with most taxa known from single bones or partial skeletons [14]. However, limited skeletal evidence has suggested a link between anatomical characteristics and distinctive tracks and particularly so-called wide gauge trackways [15]. Given this association between body proportions, skeletal anatomy and gait, any synchronous and spatiotemporal occurrence of trackways attributed to small and large titanosaurs represents a valuable source to assess scaling in this important locomotor system.

In this paper we describe a small sauropod trackway from the Late Cretaceous Fumanya tracksite (southern Pyrenees, Catalonia), which is compared with larger traces attributed to large titanosaurs from the same locality. We compare ichnological parameters that are a function of body proportions and kinematics and consider results in the context of scaling predictions, specifically geometric similarity and dynamic similarity hypotheses [16], [17]. These hypotheses state that two animals can be described as geometrically and dynamically similar if the shape and motion of one are identical to those of the other by multiplying all its linear dimensions, time intervals, and forces by different constant factors, respectively [17].

The hypotheses to be tested in the present study are that a) small and large titanosaurs possessed a geometrically similar body plan and, b) they both moved in a dynamic similar mode. The first hypothesis predicts that the relative body geometry (limb length, leg space distance from medial line, and glenoacetabular distance) were identical in both animals after multiplying each linear dimension by a constant factor. The second hypothesis predicts that both small and large animals moved with identical Froude number and relative stride lengths. We test these hypotheses on the basis of the available fossil track record by comparing track parameters of recently discovered trackways of a small titanosaur with those of corresponding larger individuals from the same locality. In addition to testing these scaling hypotheses, the trackways from Fumanya provide data from which the gait and “gauge” in titanosaurs (based on inferred trunk and appendicular limb size and proportions) can be further discussed.

### Geological Framework

**Figure 1:**
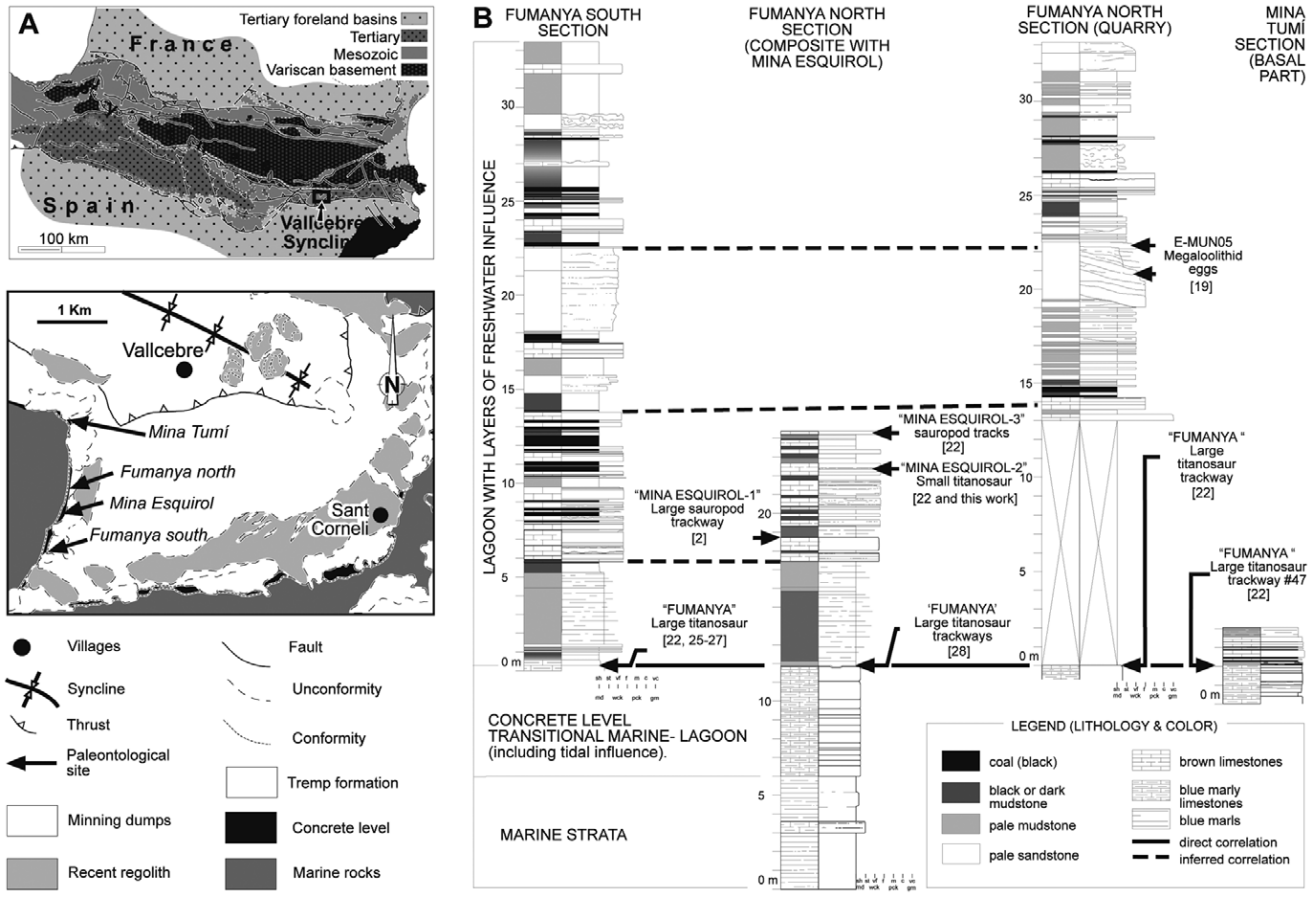
Geographic and geological setting. (A) Location of Fumanya tracksites along the Vallcebre Syncline. (B) Stratigraphical position of the sites with indication of the track-bearing levels.

The trackways included in the present study are found in Fumanya tracksites, within the Vallcebre Syncline (North of Barcelona, Catalonia, Fig. 1). Fieldwork conducted during the last decade in the Maastrichtian sediments of the Tremp Formation, yielding an important vertebrate body and trace fossils of dinosaurs, crocodiles, turtles and fishes [18]–[21]. In the Fumanya area an abundant dinosaur track record [22] is present in lower Maastrichtian beds [23]. The Fumanya tracksites crop out at four different localities (Fig. 1A and B) with tracks occurring at the base of the Tremp Formation, in at least four distinct stratigraphic levels. The trackways included in the present study are located in the “Fumanya” and “Mina Esquirol-2” track-bearing levels (Fig. 1B).

The “Fumanya” track level (on top of the so-called “concrete level”) crops out at the Fumanya South, Mina Esquirol, Fumanya North and Mina Tumí sites, along a nearly continuous (∼1.5 kilometres) track-bearing surface. Tracks occur on top of a marly limestone (5 metres thick) with overlaying centimetric overbeds. The unit contains vertebrate remains as well as abundant plant and invertebrate fossil remains. This level was deposited in a tidal mud flat recording the transition from marine to lagoonal [23], [24]. The “Mina Esquirol-2” track level crops out in the southern exposures of the Fumanya North site. The track-bearing layer comprises a medium-coarse limestone (grainstone-packstone texture) with fragments of gastropods shells (abundant cf. *Pyrgulifera* and some *Lychnus* and *Cerithium*), charophytes and undetermined plant debris. This layer is heavily bioturbated, with vertical galleries around 1 centimetre in diameter. The burrows are infilled with the lithologies of the overlying level, which is a limestone (mudstone-wackstone texture) with scarce shell fragments (only few cf. *Pyrgulifera* can be recognized). The invertebrate faunal content together with the sedimentological features indicates that these sediment packages were deposited in a lagoon environment with a freshwater influence [23], [24].

## Materials and Methods

**Figure 2:**
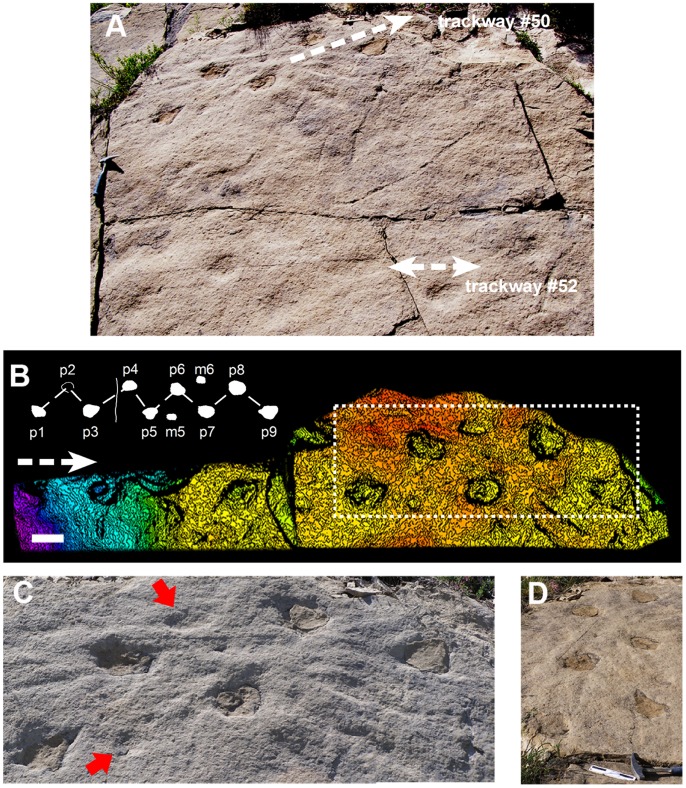
Titanosaur trackways from Fumanya localities. (A) Field picture of the small trackways (#50 and #52) from the southern edge of Fumanya North locality. Note the faint impressed tracks at the lower area. Scale: hammer (length is about 33 cm). (B) 3-D LiDAR model of the succession of tracks making up the trackway #50 with trackway sketch on the left upper corner. Scale bar: 20 cm. Dashed area corresponds to C. m: manus track; p: pes track. (C) Close-up photograph of tracks. Red arrows indicate faint manus prints in front of pes prints. (D) Partial close-up view of trackway displaying the inner trackway width feature. Progression direction is towards the top of the picture. Scale bar: 15 cm. Dashed arrow indicates progression direction in (A) and (B).

**Figure 3:**
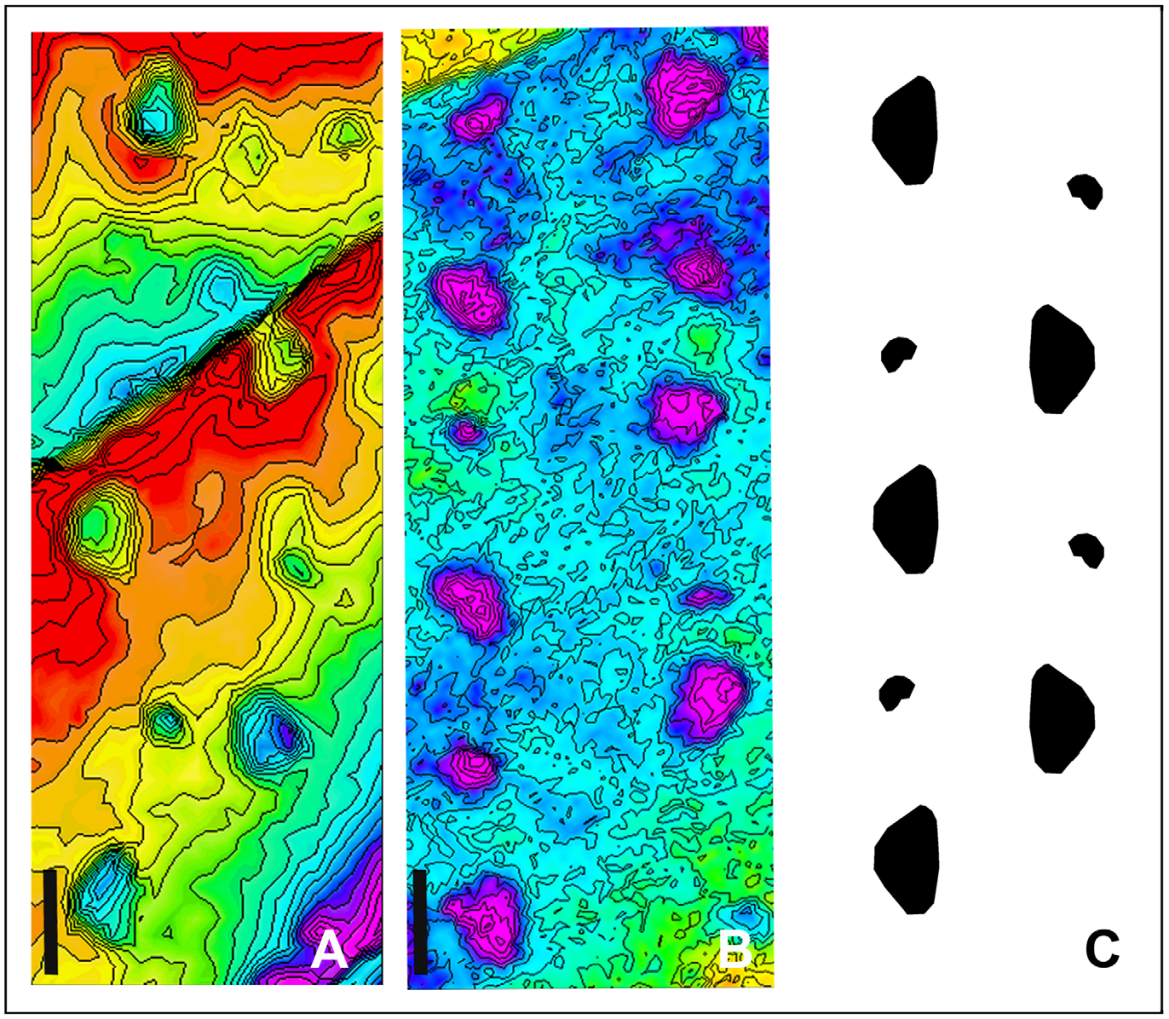
Examples of large titanosaur trackways of Fumanya. (A) trackway segment of #29 from Fumanya South locality. (B) trackway segment of #47 from Mina Tumí locality. (C) Scheme showing the trackway pattern of a large trackway based on A and B. Progression direction is towards the top of the picture. Scale bar: 1 m.

The ichnological data was obtained from eleven dinosaur trackways. Main ichnological study concentrates on one small trackway (#50, Fig. 2) located at Fumanya North. Ten additional titanosaur trackways (#9, #22, #24, #27, #28, #29, #31, #32, #37, #47; Fig. 3) located at Fumanya South, Fumanya North and Mina Tumí localities, were chosen for comparative purposes. All trackways were preliminarly mapped [22], [25]–[29]. They all have been analyzed and re-measured in detail in the present study with average values of main trackway parameters provided.

Ichnological data was acquired by using a high-resolution Light Detection and Range (LiDAR) imaging method [27]. Trackway #50 was scanned with portable a Z+F Imager 5006i laser scanner (www.zf-uk.com). All other trackways used in comparative analysis were scanned as during previous studies [27], [29] with a RIEGL LMS-Z420i 3-D LiDAR unit. The Z+F unit provides a resolution of up to <0.3 mm making it possible to scan and image the smaller, shallower tracks in #50 and #52, which was not possible using the RIEGL LiDAR unit used in our previous studies. Data from both scanners was processed identically, using the approach described in detail by Bates et al [27], [29]. Briefly, multiple scans of each outcrop were aligned to provide full 3-D coverage of the track-bearing surfaces. Merged point clouds were imported into Schlumberger’s reservoir modelling package Petrel. All measurements were made on gridded surfaces, as in Bates et al [29]. All necessary permits were obtained for the described field studies. The Departament de Cultura de la Generalitat de Catalunya issued the permission for the Fumanya localities.

In each trackway the following track and trackway parameters were measured and calculated on the 3-D model following Thulborn [30] methodology (all measurements refer to pes tracks): track length (TL; distance from the rear to the front margin of the track), track width (TW; distance from the inner to the lateral margin of the track, measured at right angle to TL), external trackway width (Twe; distance between the outer edges of the left and right tracks), stride length (SL; distance between corresponding points (center to center) in two consecutive tracks made by the same foot), pace length (PL; distance between corresponding points (center to center) in two successive tracks of alternate feet; PLa, PLb from left to right and right to left successive tracks, respectively), and pace angulation (ANG; angle made between two successive tracks). Side width (SW; width of pes tracks measured perpendicular to the long axis of the trackway), overall width (OW = Twe; distance between outer margins of pes tracks of the left and right side) and pes trackway ratio (PTR; the ratio of the side width (SW) relative to the overall width (OW) of the trackway, (SW/OW) x 100%) were measured and calculated based on Romano et al [31]. Manus-pes distance (Dm-p) was measured between the centers of the tracks of a manus-pes set (cf. [32]). The gleno-acetabular distance (Dg-a; distance between the centre of the shoulder joint and the centre of the hip joint) was calculated for an amble gait (Dg-a = Dm-p+SL), an alternate gait (Dg-a = Dm-p+SL/2), and an asynchronous gait (Dg-a = Dm-p+SL/3) for all the trackways, following the procedure of Farlow et al. [32]. Acetabulum height (cf. hip height, h) was calculated for adult titanosaurs on the basis of the h/TL ratio (h = 4.586TL), proposed by González-Riga [11].

The anatomical analysis of skeletons in titanosaurs reveals that the Dg-a/h ratio is close to 1 (Dg-a/h = 1.17 in the articulated specimen of *Ophistocoelicaudia*; Dg-a/h = 0.97 in the non-articulated specimens of *Neuquensaurus* and *Pleurocoelus*; [4]). For juvenile sauropods, we obtained the Dg-a/h ratio and h/TL ratios by using the formula proposed by González-Riga [11] on the basis of the limb bone data from a juvenile titanosauriform [33], [34], and observations and measurements taken on photographs of a mounted and articulated skeleton of such specimen (courtesy of P. Larson). The resulting Dg-a/h ratio for juveniles is 0.92. The h/TL ratio (h = 4.542TL) is very similar to that of adult titanosaurs reported in the literature [11], so we used the new value for small tiatanosaurs. The absolute speed (v) and Froude number (v^2^/gh) were calculated using the formula proposed by Alexander [16].

## Results

### Small Trackways

Trackway #50 is composed of 10 consecutive tracks over a length of 3.78 metres (Fig. 2B). Tracks are clearly distinguishable because overlaying sediment fills the tracks. The tracks are arranged in a zig-zag trackway pattern with some manus tracks located one third to halfway between two consecutive pes tracks (manus-pes distance = 21.4 cm; Dm-p/SL = 0.37; Fig. 2B, C). Pes traces are longer than wide (average TL = 16.7 cm; TW = 13 cm) and subtriangular to oval in shape. The manus are small, shallow, rounded in shape and very faintly impressed. Heteropody (manus-pes ratio) is about 1∶3. For pes tracks, the average stride length (SL) is 58.7 cm and the average pace length (PL) is 37.6 cm. The pace angulation (ANG) is 102.8° and the external trackway width (Twe) is 37.7 cm (Fig. 2D). Relative stride length (SL/h) is 0.77 (Table 1). The hip height is 75.85 cm, and the absolute speed estimate is about 0.44 m/s, with a calculated Froude number of 0.026.

**Table 1:** Trackway measurements and ratios from the trackway #50 at Fumanya North site.

SL	PLa	PLb	ANG	Twe	SW	Dm-p	SL/h	TL/SL	PTR	Dga/Twe	h/Twe	Dm-p/SL	SL/Dga
60.5	−	−	−	−	13.1	−	0.80	0.28	−	−	−	−	−
−	−	−	−	−	−	−	−	−	−	−	−	−	−
59.8	−	−	−	−	12.4	−	0.79	0.28	−	−	−	−	−
58.5	38.1	−	101.9	40.4	−	−	0.77	0.29	−	−	1.88	−	−
56.6	−	37.3	97.5	39.3	13.5	21.3	0.75	0.29	34.3	1.98	1.93	0.38	0.73
58.8	35.3	−	106.9	36.1	12.4	21.6	0.77	0.28	34.3	2.23	2.10	0.37	0.73
58.0	−	37.9	105.0	36.5	13.1	−	0.77	0.29	35.9	−	2.08	−	−
−	39.3	−	−	36.1	−	−	−	−		−	2.10	−	−
**58.7**	**37.6**	**37.6**	**102.8**	**37.7**	**12.9**	**21.4**	**0.77**	**0.28**	**34.8**	**2.10**	**2.02**	**0.37**	**0.73**

SL, PL, and Twe in cm. ANG in degrees. PTR in %. Average values in bold.

Trackway #50 shows a mean PTR value of about 34% indicating that gauge condition clearly lies in the wide-gauge category (≤36%) rather than that of medium or narrow category (≥50%). Whilst we recognize the wide- to narrow-gauge probably represents a false dichotomy or oversimplification (variation in gauge width is probably more of a continuum; e.g. [31], [35]), we nevertheless consider it valid to categorize them as wide-gauge trackways.

On the same surface as trackway #50, there is an additional trace, here referred to as trackway # 52 (Fig. 2A). This is composed of six faintly impressed pes prints that can be observed under good light conditions. They comprise a trackway of about 2.25 metres. They are arranged in a zig-zag trackway pattern and are oval in shape. They probably represent another small sauropod trackway but because of the faint preservation we did not take trackway measurements or make further calculations. Trackway #50 and tentatively trackway #52 are assigned to sauropods because of the general trackway pattern and the track morphology. They display various features that support a sauropod-type affinity, notably tracks arranged in a zig-zag configuration pattern, and low pace angulation value [30]. More specifically there is good evidence to suggest trackway #50 can be attributed to a titanosaur because of its wide gauge condition [12] and other parameters such as the Dm-p/SL ratio. Previous works have also supported the conclusion that Fumanya trackways were produced by derived titanosaurs [12], [22], [25]–[29]. Their close location, size, and general appearance (track size and trackway pattern) suggest that animals of similar size produced both trackways (#50 and #52).

### Large Trackways

The titanosaur trackways selected for comparative purposes represent complete sequences of pes and manus tracks (e.g. trackway #47 is composed of about 73 pes and 25 manus tracks) arranged in a zig-zag pattern and comprising trackways of up to 60 metres (Fig. 3). The pes tracks representing average morphology (from trackways #29 and #47) are longer than wide (average TL∼ 72 cm; TW = 62 cm) and subtriangular in shape. When preserved, they depict four outward rotated claw marks at the deeper impressed anterior margin and a narrow heel impression in the posterior, shallow edge (Fig. 1.11 in [22] and Fig. 5 in [29]). All the pes tracks correspond to trackmakers of similar size (TL ranges from 56.14 to 72.66 cm; Table 2). The manus tracks are smaller and deeper impressed with a U-shaped outline. They are located one third to one half away from the center of the preceding pes track (Dm-p/SL ratio ranges from 0.28 to 0.50). Heteropody (manus-pes ratio) is 1∶3. All the trackways present a low pes trackway ratio (PTR = 24.24 to 31.16%) indicating very wide- to wide-gauge trackmakers (Table 2). Relative stride lengths (SL/h) are about 0.66–0.84. Estimated values for absolute speeds are low and range from 0.69 to 1.02 m/s. The respective Froude numbers range from 0.015 to 0.035.

**Table 2:** Average measurements and ratios of large trackways from Fumanya South and Mina Tumí localities.

Trackway	TL	SL	Twe	SW	Dm-p	SL/h	TL/SL	PTR	Dg-a/h	Dg-a/Twe	h/Twe	Dm-p/SL	SL/Dg-a	Fn
**#9**	61.01	222.80	192.29	56.01	62.2	0.80	0.28	29.97	1.01	1.51	1.48	0.28	0.78	0.029
**#22**	59.11	222.63	147.43	43.34	77.8	0.82	0.27	29.39	1.10	2.03	1.84	0.35	0.74	0.032
**#24**	65.64	213.07	223.02	57.88	86.6	0.71	0.31	25.93	0.99	1.33	1.35	0.41	0.71	0.020
**#27**	56.14	200.84	192.89	53.66	88.7	0.78	0.28	28.09	1.13	1.52	1.34	0.44	0.69	0.027
**#28**	68.42	205.86	200.63	61.66	89.4	0.66	0.33	30.72	0.94	1.47	1.57	0.43	0.70	0.015
**#29**	71.24	246.89	168.97	52.57	126.5	0.76	0.29	31.16	1.15	2.24	1.94	0.50	0.67	0.025
**#31**	64.80	250.79	177.33	49.15	89.5	0.84	0.26	27.75	1.14	1.90	1.68	0.35	0.74	0.035
**#32**	66.32	235.05	196.65	47.59	90.1	0.77	0.28	24.24	1.07	1.65	1.55	0.38	0.72	0.026
**#37**	62.47	241.67	187.12	50.10	99.3	0.84	0.26	26.87	1.18	1.84	1.53	0.42	0.71	0.035
**#47**	72.66	222.91	225.65	59.10	110.0	0.67	0.33	25.88	0.99	1.48	1.48	0.48	0.68	0.016
**Average**	64.78	226.25	191.20	53.11	92.01	0.76	0.29	28.00	1.07	1.70	1.57	0.40	0.71	0.026
**SD**	5.23	17.04	23.43	5.69	17.36	0.07	0.03	2.30	0.08	0.29	0.19	0.07	0.03	0.007

TL, SL, Twe, SW, and Dm-p in cm. PTR in %. Dg-a values are shown for an amble gait (Dg-a = Dm-p+SL; [31]) as those that more closely approximates to the empirical relationship of Dg-a/h ratio obtained by [4] for adult titanosaurs.

## Discussion

### Hypothesis (a): Titanosaur Body Plan and Geometrical Similarity

The geometric similarity hypothesis stated that two bodies are geometrically similar if one could be made identical to the other by multiplying all length dimensions by the same factor [16], [17]. In animal biomechanics scaling is typically calculated relative to body mass, such that geometrical similarity in linear dimensions describes a situation in which lengths are proportional to body mass^0.33^. Body mass is notoriously difficult to calculate accurately for extinct animals like dinosaurs even when near-complete skeletons are available (e.g. [36]), and as yet no validated method has been derived to extrapolate masses from footprints. Here we diagnose geometric similarity from trackways using the relative proportions of body segment lengths (e.g. [37]); consistency relative to trackway geometry is interpreted as diagnostic of isometry in linear body proportions of the trackmakers. We recognise, however, that determining body dimensions from tracks must be catiously undertaken, given track size can easily be a function of substrate type and conditions (at the time of track formation), with variation in trackmaker size a function of rheology [38]. It is worth to underscore that the studied sample refers to two discrete populations of one small versus ten large titanosaurs; thus, it is not a continous distribution of sizes (i.e., a small trackway being compared to a population of large trackways).

In the present study the comparison of the values of gleno-acetabular distance (Dg-a) and hip height (h) of the small trackway (#50) with respective measurements of large trackways reveals that some of the ratios show very similar values (Table 2). The relationship between the trunk length and the hip height of the small trackmaker (Dg-a/h = 1.04) lies within the respective standard desviation values of the large trackmakers (mean Dg-a/h ratio ± SD is 1.07±0.08). This suggests that, on the basis of ichnological evidence, the trunk length (cf. gleno-acetabular distance) was proportionally similar to hindlimb length (cf. hip height) in both the small and large titanosaurs and that scaling was close to geometrical similarity.

In respect to the body width, ichnological studies based on tracksites containing both small and large trackways reveal that juvenile and adult trackmakers of some sauropods, probably basal macronarians or “diplodocids”, would have produced narrow-gauge trackways [39]. Medium to wide-gauge juvenile trackways have been attributed to “brachiosaurids” or “titanosaurids” [35]. Wilson & Carrano [12] suggested that skeletal morphology of titanosaurs is responsible for gauge differences and predicted that derived titanosaurs (∼saltasaurids or titanosaurids *sensu* [40]) possessed particular anatomical characteristics in their limb structure allowing the close adscription with wide gauge trackways. Wilson & Carrano [12] went on to suggest that trackway gauge disparity does not result from differences in body size and therefore individuals of different sizes could not produce both narrow- and wide-gauge trackways. Lockley et al. [10], [41], [42] proposed that gauge differences are in part a function of size and growth and pointed that gauge of juveniles appears to have ranged from narrow gauge to moderately wide. At the Fumanya tracksites, where more than 40 trackways have been reported, there is no evidence of narrow- or medium-gauge trackways [22], [26], [29] and small and large titanosaurs produced wide to very wide-gauge trackways, respectively. PTR values are low (mean PTR ± SD for large trackways is 28.00% ±2.30; and 34.8% for the small trackway; Table 2) and are consistent with the hypothesis that both small and large titanosaurs were wide-gauge trackmakers (cf. [12]).

Comparison of other relative body parameters such the gleno-acetabular distance and the trunk width or hip height relative to the trunk width (i.e. Dg-a/Twe and h/TWe, respectively) of small and large trackways indicate a remarkable similarity in trunk and limbs arrangement, in spite of some minor differences. For example, the Dg-a/Twe ratio of the small trackway (Dg-a/Twe = 2.10) lies within two SD of the large titanosaurs’ sample (mean Dg-a/Twe ± SD = 1.70±0.29). In a similar way, the h/Twe ratio of the small trackway lies within three SD of the large titanosaurs’ sample (mean h/Twe ± SD = 1.57±0.19). Such minor variations are probably due to the fact that, even though they both were wide-gauge trackmakers, the “wide condition” in small titanosaurs (high PTR values) might not be so exagerated as in the large individuals (very high PTR values), as suggested by Lockley et al. [10], and this influenced other relative body parameters. In any case and in spite that a larger sample size is probably needed, PTR values below 36% support a clear wide-gauge condition in both small and large titanosaurs. This implies that the general titanosaur *bauplan* in regards to the pelvic girdle and limb structure, that is associated with wide-gauge locomotion, was probably independent of size. Therefore, we conclude that small and large titanosaurs had similar body (trunk and limbs) proportions regardless of their size; thus, they scaled close to geometric similarity.

This finding is in agreement with previous works reporting isometric growth in appendicular limbs of most sauropod groups [33], [43], [44]. However, it is worth to underscore that from the data selected for the present study we cannot assure that differences in size observed in the trackways refer to growth stages. Juvenile individuals of the same species that produced the large trackways may have produced the small traces; alternatively, small and large trackways may represent individuals of different-sized species. With this in mind the authors acknowledge the limits of their inferences, which will refer to body plan, limb arrangement, and locomotion of different-sized titanosaurs regardless their taxonomic level.

### Hypothesis (b): Titanosaur Gaits and Dynamic Similarity

Dynamic similarity hypothesis predicts that bodies that are comparable in shape, but different in size, move in dynamically similar ways if they have equal values of Froude number and relative stride length [16], [17]. In consequence, they will produce similar trackway patterns due to their comparable motion and body plans (i.e. limbs and trunk proportions). In the case of sauropod dinosaurs, the relative proportions in gleno-acetabular distance (the distance between pelvic and scapular girdles) and length and excursion angles of the fore- and hindlimbs limit their movements [6] in three main gaits: a) an amble pace gait; b) an alternate pace gait and c) an asynchronous pace gait [32]. On trackways, distinct gaits are expected to produce differences in stride length and manus-pes distance as well as in calculation of the glenoacetabular distance.

**Figure 4:**
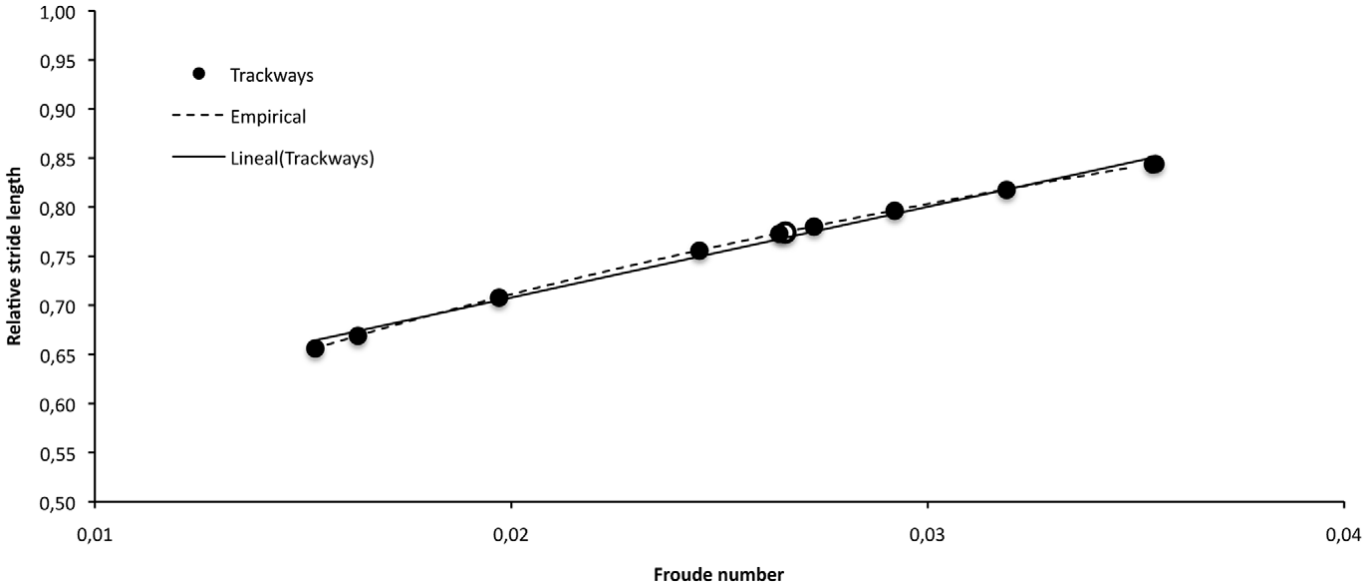
Graph showing the relative stride length against Froude number, for Fumanya titanosaurs and also the empirical SL/h≅2.3(v^2^/gh)^0.3^ relationship [**16**]. White circle refers to the small trackway (#50).

From the studied trackways several parameters and ratios indicate that both small and large titanosaurs moved in a dynamically similar fashion, with a similar gait. First, titanosaurs producing both the small and the large trackways present very similar Froude numbers (mean Fn ± SD = 0.026±0.007 for large trackmakers; Fn = 0.026 for the small trackmaker) and have low values for relative stride lengths (mean SL/h ± SD = 0.76±0.07 for large trackmakers; SL/h = 0.77 for the small trackmaker), clearly indicating a dynamically similar walking gait. Plotted data for all the trackways indicates that a strong positive correlation exists between Froude number and relative stride length parameters (R^2^ = 0.99206) in Fumanya titanosaurs, and that this relationship scales very close to the empirical prediction of dynamic similarity (Fig. 4). Second, we calculated the three possible gaits for all trackmakers following considerations of Farlow *et al.* [32] and assuming the Dg-a/h relationship measured in reconstructions of sauropod skeletons (which is close to 1 for titanosaurs; see Materials and methods). We conclude that in all the cases, among the three calculated Dg-a (amble, alternate, asynchronous; [32]) the one that better fits with the Dg-a≥h value obtained from skeletons is that corresponding to an amble pace gait, where the ipsilateral limbs move almost synchronously. Third, this particular mode of locomotion is reflected in other ratios calculated from trackway parameters. For example, in the small trackmaker the values of the relationship between the length of the hindfoot and the excursion angle of the hindlimb (TL/SL = 0.28) or the excursion angle of the hindlimb relative to the body length (SL/Dg-a = 0.73) lie within the range of the large titanosaurs’ sample (mean TL/SL ± SD = 0.29±0.03; mean SL/Dg-a ±SD = 0.71±0.03). Therefore, present data indicate that small titanosaurs walked in the same general manner as large titanosaurs producing geometrically comparable trackways. This may concur with Carrano [45] who stated that juvenile dinosaurs represented the locomotor morphology (and therefore the same gait, stance and gauge) of adult dinosaurs.

### Conclusions

The comparative analysis of the small and large sauropod trackways from the Late Cretaceous Fumanya tracksite (southern Pyrenees, Catalonia) in the context of the geometric and dynamic similarity hypotheses provides significant information on gait, gauge and body proportions of the respective trackmakers. Ichnological ratios reveal similarities in gait, body and limb proportions suggesting that trunk and limbs’s lengths of small titanosaurs were similar to that of large individuals. As a consequence, similar Froude numbers and relative stride lengths in both small and large trackmakers indicate that titanosaurs moved in a dynamically similar way regardless their body size. Finally, the wide condition reported in trackways of small and large titanosaurs implies that they both possessed the same pelvic girdle and limb structure, which is associated with wide-gauge locomotion.
