# Correction: Dynamic Similarity in Titanosaur Sauropods: Ichnological Evidence from the Fumanya Dinosaur Tracksite (Southern Pyrenees)

**DOI:** 10.1371/journal.pone.0219809

**Published:** 2019-07-10

**Authors:** Bernat Vila, Oriol Oms, Àngel Galobart, Karl T. Bates, Victoria M. Egerton, Phillip L. Manning

In the Materials and Methods, there is an error in the equation for the calculation of an asynchronous gait. Please view the complete, correct equation here: Dg-a = Dm-p+3/4SL
